# Development of a fast and easy method for *Escherichia coli* genome editing with CRISPR/Cas9

**DOI:** 10.1186/s12934-016-0605-5

**Published:** 2016-12-01

**Authors:** Dongdong Zhao, Shenli Yuan, Bin Xiong, Hongnian Sun, Lijun Ye, Jing Li, Xueli Zhang, Changhao Bi

**Affiliations:** 1 Tianjin Institute of Industrial Biotechnology, Chinese Academy of Sciences, Tianjin, 300308 China; 2 Key Laboratory of Systems Microbial Biotechnology, Tianjin Institute of Industrial Biotechnology, Chinese Academy of Sciences, 32 West 7th Ave, Tianjin Airport Economic Park, Tianjin, 300308 China

**Keywords:** CRISPR/Cas9, *E. coli*, Genome editing

## Abstract

**Background:**

Microbial genome editing is a powerful tool to modify chromosome in way of deletion, insertion or replacement, which is one of the most important techniques in metabolic engineering research. The emergence of CRISPR/Cas9 technique inspires various genomic editing methods.

**Results:**

In this research, the goal of development of a fast and easy method for *Escherichia coli* genome editing with high efficiency is pursued. For this purpose, we designed modular plasmid assembly strategy, compared effects of different length of homologous arms for recombination, and tested different sets of recombinases. The final technique we developed only requires one plasmid construction and one transformation of practice to edit a genomic locus with 3 days and minimal lab work. In addition, the single temperature sensitive plasmid is easy to eliminate for another round of editing. Especially, process of the modularized editing plasmid construction only takes 4 h.

**Conclusion:**

In this study, we developed a fast and easy genome editing procedure based on CRISPR/Cas9 system that only required the work of one plasmid construction and one transformation, which allowed modification of a chromosome locus within 3 days and could be performed continuously for multiple loci.

**Electronic supplementary material:**

The online version of this article (doi:10.1186/s12934-016-0605-5) contains supplementary material, which is available to authorized users.

## Background

Microbial genome editing is for site-specific chromosome modification, which is one of the most useful techniques in research and plays an important role in metabolic engineering and molecular biology research. Current methods include homologous recombination-based gene targeting [1–3], zinc finger nucleases (ZFNs) [4] and transcription activator-like effector nucleases (TALEN) [5, 6]. However, these techniques are often inefficient, time consuming, laborious, or expensive to use. Thus, a rapid and simple method capable of performing precise genome editing with minimal cost remains to be desired.

The clustered regularly interspaced short palindromic repeats (CRISPR)/CRISPR-associated protein (Cas) system is an RNA-guided immune system in many bacteria [7]. This system is able to recognize and generate double-strand breaks (DSBs) at target sequence. Following the DSB event, native DNA repair will be initiated through non-homologous end joining or homologous recombination in the presence of an exogenous homologous donor sequence [8]. The only restriction for designing a CRISPR/Cas9 guiding sequence is need for a protospacer adjacent motif (PAM) close to genomic target site [9]. The typeII CRISPR/Cas9 system of *Streptococcus pyogenes*, which requires a mature CRISPR RNA (crRNA), a trans-activating crRNA (tracrRNA) and a DNA endonuclease Cas9 has attracted significant attention and been harnessed for targeted genome editing in different organism, both Eukaryotes and Prokaryotes, such as *E. coli* [10–12], human [13], mice [14] and zebrafish [15]. Thus far, the CRISPR-Cas9 system is a powerful and revolutionary tool for genome editing.

The CRISPR/Cas9 system has been widely used for eukaryotic genomes but its applications in bacterial genomes were less studied. CRISPR/Cas9 system was firstly exploited for bacterial genomic editing by Jiang et al., the work was a huge breakthrough for microbial genomic editing, however, high editing efficiency in *E. coli* was not achieved [10]. Recently, researchers achieved very good results in development of Cas9 based gnomic editing techniques [10–12, 16]. While these methods improved the genome editing techniques, some aspects could be further improved, such as editing efficiency, lab hours, convenience of usage and standard protocols. To tackle these problems, we started the work of development of a simple and fast method for bacterial genome editing with high efficiency. For this purpose, multi-plasmid system was experimented and found to be relatively laborious and time consuming. Low editing efficiency was observed with the plasmid/linear DNA system, especially with wild type and *E. coli* cell factory strains. This was probably due to their strong restriction systems which degrade the linear donor DNAs before integration into chromosome [17]. To avoid these drawbacks, our strategy was designed to edit genome with only one plasmid, which carried all functional parts to make the technique very easy and fast to practice and achieve high efficiency.

## Methods

### Bacterial cultivation

*Escherichia coli* MG1655 and *DH*_*5*_ *α* were grown at 30 °C in lysogeny broth (LB, 1% (w/v) tryptone, 0.5% (w/v) yeast extract, 1% (w/v) NaCl). Kanamycin (50 mg/L for *E. coli*) and Ampicillin (100 mg/L for *E. coli*) were added to the medium as appropriate. 1% (w/v) glucose was added to the culture for Cas9 repression and l-arabinose (2 g/L final concentration) was used for Cas9 induction when necessary. IPTG and X-gal for blue/white selection were added at concentrations of 0.1 mM and 40 µg/mL, respectively [12].

*Escherichia coli* MG1655 were grown in 5 mL LB cultures at 30 °C to an OD600 of 0.6 and then made electrocompetent by concentrating 100-fold and washing three times with ice-cold 10% glycerol. 10–100 ng of PCR target plasmid was used for electroporation, shocked cells were added to 1 mL LB, incubated 1 h at 30 °C, and then plated on LB petri dishes carrying appropriate antibiotics.

### Plasmid construction

The Cas9 gene from Cas9 expression plasmid (Addgene reference number: 42876), gRNA without N20 (5′- GTTTTAGAGCTAGAAATAGCAAGTTAAAATAAGGCTAGTCCGTTATCAACTTGAAAAAGTGGCACCGAGTCGGTGCTT-3′) and its promoter (5′- CTAGGTTTATACATAGGCGAGTACTCTGTTATGGAGTCAGATCT-3′) which were all directly synthetized. pKD46 modular (contains: exo, bet, gam, rep and arabinose operon) was used for plasmid construction as backbone.

pCas9 series and pRed_Cas9 series other than pRed_Cas9_recA_Δ*poxb*41 and pRed_Cas9_recA_Δ*lacZ*41 were assembled with the Gibson method, of which DNA oligo primers were designed with j5 and Device Editor [18]. DNA templates were PCR-amplified with Phusion polymerase (NEB) or directly synthesized. PCR products were gel purified, digested with DpnI before Gibson amplification.

For modular design, the pRed_Cas9_recA_Δpoxb300 plasmid was used as template for the PCR amplification of target modules. Homologous arms of H1 and H2 were obtained from genomic DNA of *E. coli* MG1655. pRed_Cas9_recA_Δ*poxb*41, pRed_Cas9_recA_Δ*poxb*::*rfp*41 and pRed_Cas9_recA_Δ*lacZ*41 were assembled using the modular assembly method.

### Strain and plasmid availability

The plasmids used in this study are listed in Table 1. *E. coli* DH5α was used as a cloning host, for fast performance, reaction mix assembled using the modular assembly method was directly transformed into *E. coli* MG1655. *E. coli* MG1655 was used in the genome engineering procedures. The coding sequences of all plasmids and modular parts developed here are physically available from the authors.

**Table 1:** The plasmids used for this work

Plasmids	Relative characteristics	Resources
pCas9	Cas9	[10]
pKd46	Exo, bet, gam, rep and arabinose operon	[3]
pAra_Cas9_Δpoxb300	Arabinose operon, Cas9, gRNA with N20 and with homologous arms of about 300 bp for *poxb*	This work
pRed_Cas9_Δ*poxB*300	Derived from pKD46, exo, bet, gam, arabinose operon, Cas9, gRNA with N20 and with homologous arms of about 300 bp for *poxb* deletion	This work
pRed_Cas9_Δ*poxb*::*rfp*300	Derived from pRed_Cas9_Δ*poxB*300, and with homologous arms of about 300 bp with *rfp* replace *poxb*	This work
pRed_Cas9_Δ*poxb*100	Derived from pRed_Cas9_Δ*poxB*300, and with homologous arms of about 100 bp for *poxb* deletion	This work
pRed_Cas9_Δ*poxb*50	Derived from pRed_Cas9_Δ*poxB*300, and with homologous arms of about 50 bp for *poxb* deletion	This work
pRed_Cas9_recA_Δ*poxb*300	Derived from pRed_Cas9_Δ*poxB*300, *recA*	This work
pRed_Cas9_recA_Δ*poxb*41	Derived from Red_Cas9_recA_Δ*poxb*300, and with homologous arms of about 41 bp for *poxb* deletion	This work
pRed_Cas9_recA_Δ*poxb*::rfp41	Derived from Red_Cas9_recA_Δ*poxb*300, and with homologous arms of about 41 bp for rfp replace poxb	This work
pRed_Cas9_recA_Δ*lacZ*41	Derived from Red_Cas9_recA_Δ*poxb*300, and with homologous arms of about 41 bp for *lacZ* deletion	This work

### Genome editing procedure

A pair of 24 nt oligos and a pair of 86 nt oligos were designed to target genomic locus for editing. A simple DNA annealing procedure was used to obtain the N20 part and donor DNA part, which were assembled with Part1 and Part2 with a Golden Gate assembly reaction to the editing plasmid. Then the plasmid was transformed into *E. coli*, followed by plating on LB+ Kan+ 1% (w/v) glucose plate, and culturing at 30 °C. The resulting strain was grown in LB+ Kan for 2 h before 2 g/L l-arabinose induction for Cas9 and gRNA expression. After culturing of 6 h, the culture was plated on LB+ Kan+ l-arabinose agar plates, and the colonies were analyzed by colony PCR with a forward primer upstream of the left homology arm and a reverse primer downstream of the right homology arm. Colonies with expected PCR product were subjected to verification by DNA sequencing for further confirmation. At last, the temperature sensitive editing plasmid is cured by growing the edited strains at 37 °C overnight. A detailed genome editing protocol is provided in supplemental file (Additional file 1).

### Editing efficiency calculation

For editing efficiency calculation, cell culture after induction process was diluted 100 fold, of which 20 μL was plated on LB dishes for proximate colony counts. Colonies was screened by PCR and DNA sequencing to indentify correctly edited clones. With each experiment, 48 colonies are screened by colony PCR and the correct colonies were used for further verification by DNA sequencing. The editing efficiency equals the percentage of the correct colonies in all analyzed colonies. All experiments were repeated three times to obtain the means and standard variations.

When *lacZ* gene was targeted, cells were plated on IPTG and X-gal plates. The apparent editing efficiency was ratio of white colonies of total colonies. For further verification, white colonies were identified by colony PCR and DNA sequencing to obtain the real editing efficiency, which equaled ratio of the correctly edited colonies of total colonies.

## Result

### CRISPR/Cas9 based chromosomal gene deletion of *poxB* with one plasmid construction and one transformation

**Fig. 1:**
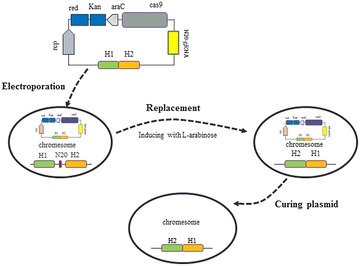
The procedure of genome editing with one plasmid and one transformation. The first step is to assemble the editing plasmid. For the second step, the plasmid is transformed into *E. coli*, followed by plating on LB+ Kan+ 1% (w/v) glucose plate, and grew at 30 °C. For the third step, the resulting strain is grown in LB+ Kan for 2 h before induction with 2 g/L l-arabinose for Cas9 and gRNA expression. After culturing of 6 h, the culture is plated on LB+ Kan+ l-arabinose agar plates. Last step, the temperature sensitive editing plasmid is cured by growing the edited strains at 37 °C overnight

In this research we designed a genome editing method with one plasmid construction and one transformation. The CRISPR/Cas9 genomic editing system includes two parts, Cas9 and gRNA, simultaneous expression of which causes cleavage of bacterial genome, leaves blunt ends and causes cell death [19]. For this reason, Cas9 or gRNA should be repressed before genome editing event. In our preliminary studies, lac-derived promoter was unsuccessful as an inducible system for cas9 and gRNA expression due to its leaky expression. Thus, the glucose repressed, l-arabinose induced pBAD promoter was employed to express Cas9. With this strategy, the primitive one-plasmid system was designed to include functional parts of Cas9, gRNA and the temperature-sensitive replicon repA101ts [3]. The one plasmid editing procedure is illustrated in Fig. 1.

A non-essential gene *poxB*(GeneID: 946132) was selected as a target gene in *E. coli* MG1655 for genome editing [20]. An editing plasmid, pAra_Cas9_Δpoxb300, was constructed to delete 513 bp chromosomal fragment within *poxB*, which employed 300 bp homologous arms to recombine with the target locus. The plasmid was electroporated into *E. coli* MG1655 to initiate genome editing. After culturing and induction with l-arabinose (Fig. 1), the culture was diluted 100 fold, of which 20 μL were plated to obtain edited strains. A few hundred colonies were obtained. For the control experiment, cells were cultured without l-arabinose induction, thus, little Cas9 was expressed. The control group had many more colonies appeared on plate to form a bacterial meadow. This result demonstrated that Cas9 and gRNA were successfully expressed and functional. After arabinose induction, gRNA guided Cas9 to have killed most bacterial population that did not undergo *poxB* editing. By colony PCR and DNA sequencing, 20.8 ± 8.8% random picked colonies from experiment group were determined to have been successfully edited, while no correctly edited ones from control group. This result demonstrated that our genome editing method was functional as expected.

### λRed recombinases improved editing efficiency

**Fig. 2:**
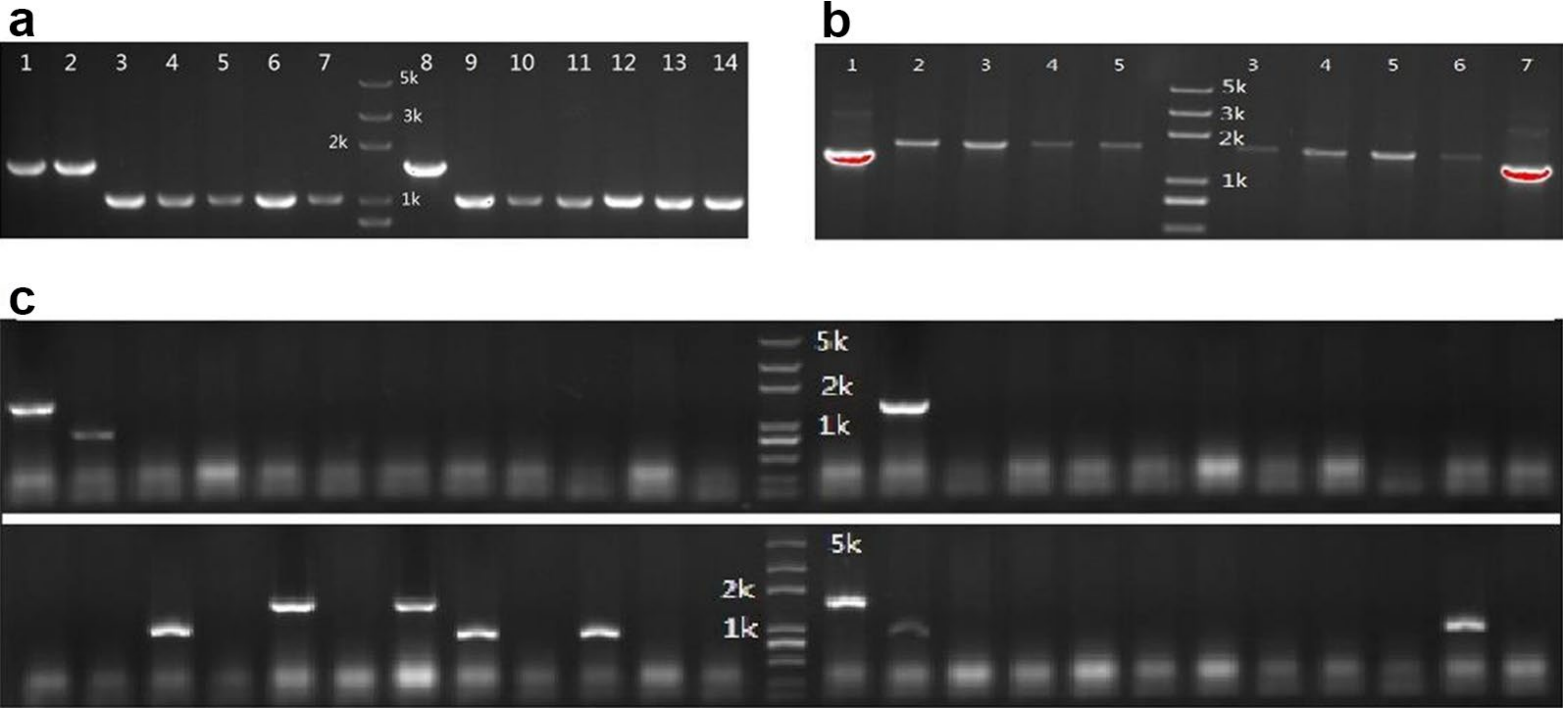
Agarose gel electrophoresis of colony PCR for *poxb* editing. **a** *poxb* gene knock out with 513 bp deletion: *lanes 1*,*2* and *8* were control group samples, others were experimental group samples. Colony PCR products of edited *poxb* gene were 1008 bp, and original were 1521 bp. **b** *poxb* replacement by *rfp*: *lanes 1* and *7* were control groups, others were experimental groups. Colony PCR products of edited *poxb* gene were 1823 bp, and original were 1521 bp. **c** *poxb* disruptions with homologous arms of 41 bp with both RecA and *Red* recombinases: all the bands were experimental group samples. Colony PCR products of edited *poxb* gene were 1008p

To increase editing efficiency, we integrated λRed recombinases into the CRISPR/Cas9 plasmid, pRed_Cas9_Δ*poxB*300. This new one-plasmid system was tested to implement 513 bp chromosomal deletion of *poxb* gene with 300 bp homologous arms. Colonies from experimental group and control group were randomly picked for colony PCR identification with primer pairs pkd_poxb_F and pkd_poxb_R. agarose gel electrophoresis of colony PCR was shown in Fig. 2a, that all experimental group colonies showed the expected shortened length after editing procedure. On the contrary, all colony PCR bands of control group showed original length. Sequencing data further proved that shortened bands were edited gene loci. Based on the results, although not all colonies were identified with PCR, 100% genome editing efficiency was achieved from randomly picked colonies. In addition, 1400 bp chromosomal DNA of the *poxB* gene was also experimented to be deleted with 100% efficiency.

The one-plasmid genome editing system was then carried out for chromosomal gene replacement experiment. Plasmid pRed_Cas9_Δ*poxb*::*rfp*300 was constructed for replacement of *poxb* with *rfp* gene. In this study, 513 bp of *poxb* was replacement by an 815 bp fragment, which consisted of *rfp* gene and SD sequence. A high editing rate of 100% was also obtained, part of the agarose gel electrophoresis of colony PCR was shown in Fig. 2b. The results demonstrated that with supplementation of λRed recombinases, the one plasmid strategy worked with 100% efficiency either with genomic knockout or replacement. And the target deletion length did not affect editing efficiency at least within 1500 bp range, which is long enough for normal gene knockout experiments.

### Genomic editing efficiency decreased with shorter homologous recombination arms

To simplify plasmid construction, different length of homologous recombination arms were tested to find the shortest that were functional. Homologous arms of around 300, 100, and 50 bp were chosen to test their editing efficiency. Plasmids pRed_Cas9_Δpoxb100 and pRed_Cas9_Δpoxb50 were constructed to carry out the experiments. For each length, random colonies from the plates were identified with colony PCR. The results were illustrated in Table 2. With homologous arms of 101 bp and 101 bp, the editing efficiency dropped to 69.3 ± 1.17%. And with 51 bp of homologous arms, we failed to obtain any successful edited clones from 50 colonies. This result indicated with current system, the editing efficiency declined rapidly with shorter homologous arms. Especially for the length of homologous arms less than 50 bp, the editing efficiency dropped to 0%.

**Table 2:** The editing efficiency and colony number at *poxB* locus with different length of homologous arms

Homologous arm length	Proximate colony number	Editing efficiency
297 bp plus 298 bp	500	100 ± 0%
101 bp plus 101 bp	150	69.3 ± 1.17%
51 bp plus 51 bp	50	0

### Addition of *recA* enabled short homologous recombination arms for successful genomic editing

For our one-plasmid genomic editing system, the most laborious part of work was plasmid construction. If the homologous arms were short enough, the two homologous arms could be assembled by primer embedding, instead of PCR and ligation. To simplify plasmid construction, it was important to make shorter homologs arms work for this method.

Reports have shown that recA improved red/ET recombination efficiency [21]. To increase editing efficiency, *recA* gene was added to the one plasmid system to create pRed_Cas9_recA_Δ*poxb*41 plasmid which carried λRed recombinases, CRISPR/Cas9 system, *recA* and two homologous arms of 41 bp. An editing efficiency of 13.8 ± 7.9% was achieved in *E. coli* MG1655 with *poxB* gene deletion. Parts of the agarose gel electrophoresis of colony PCR is shown as Fig. 2c. For gene replacement, pRed_Cas9_recA_Δ*poxb*::*rfp*41 was constructed for replacement of poxb with *rfp* gene. With 41 bp homologous arms, an editing efficiency of 16.7 ± 3.8% was achieved. The editing efficiency with 41 bp homologous arms is acceptable for genome editing experiment, since even with this efficiency, at least one successfully edited strain could be obtained from seven to eight colonies. Furthermore, colony PCR screening is not laborious and is to be performed even with high editing efficiency experiments.

### One-plasmid method enabled convenient multi-round genome editing

To demonstrate the application of one-plasmid system for multiple round editing, *lacZ*(Gene ID: 945006) gene was selected for target of the second round for the convenience of color indication [12]. After MG1655*Δpoxb* was obtained, editing plasmid pRed_Cas9_recA_Δ*poxb*41 was cured by growing colonies overnight at 37 °C. pRed_Cas9_recA_Δ*lacZ*41 was constructed to delete 1 kb of *lacZ* gene and transformed into MG1655*Δpoxb*. After induction process, the culture was diluted tenfold, of which 20 μL were plated, and editing efficiency was calculated and showed in Table 3. Apparent efficiency represented percentage of white colonies in total colonies on plate, which showed phenotype of Δ*lacZ*. The editing efficiency was percentage of the colonies confirmed with PCR in total colonies. Interestingly, PCRs of some colonies turned out to have neither edited band nor original band, which was consistent with *poxb* editing situation. These colonies contributed to apparent edition efficiency but not editing efficiency. In addition, all the colonies verified for *lacZ* editing were also subjected for colony PCR of *poxb* and confirmed to keep the *poxb* deletion. This result proved genomic stability of editing locus during multiple rounds by this method.

**Table 3:** Efficiency of the two round consecutive editing with one-plasmid method

	Apparent efficiency	Editing efficiency
First round at *poxB*	Not determined	13.8 ± 7.9%
Second round at *lacZ*	89.7 ± 0.9%	19.9 ± 4.1%

### Development of modularized assembly strategy for plasmid construction

**Fig. 3:**
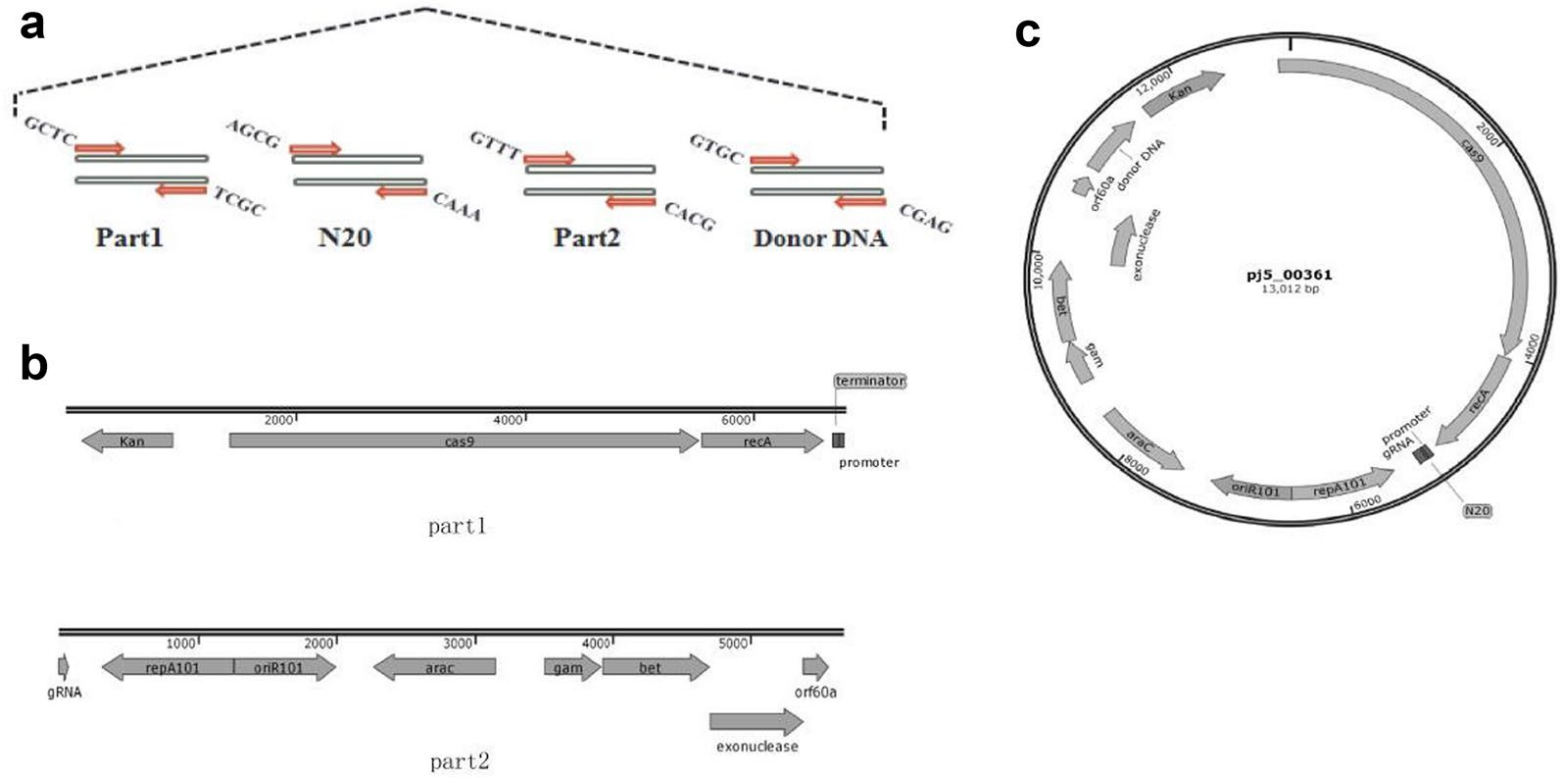
Illustration of modularized assembly strategy. **a** Editing plasmid modular parts with optimized 4 nt TypeIIS resection enzyme linkers; **b** maps of fixed backbone modular part1 and part2; **c** map of assembled pRed_Cas9_recA_Δpoxb300 from modular designed parts

Using homologous arms of around 40 bp, we were able to design a rapid method of plasmid construction using a modular approach with Golden Gate assembly strategy [22]. The modularized plasmid (pRed_Cas9_recA_Δ*poxb*41) was divided into four parts: part1, part2, N20 and donor DNA. The four parts were designed for each to carry a pair of 4 bp TypeII restriction enzyme linkers as illustrated in Fig. 3a, which were tested and optimized for best assembly efficiency.

Modular Part1 and part2 were the basic compositions for plasmid construction, which were ready made for editing of different loci, as showed in Fig. 3b. Part1 consisted of a resistance gene, an arabinose-inducible pBAD promoter with Cas9 and *recA*, a terminator, and a constitutive promoter for gRNA. Part2 consisted the temperature-sensitive replicon repA101ts, gRNA without N20 leading sequence, *exo, bet, gam, rep* and *araC*. N20 parts and donor DNA parts were annealed from synthesized DNA oligos. A pair of 24 nt oligos and a pair of 86 nt oligos were needed for editing of one locus. Only a simple DNA annealing experiment and a Golden Gate assembly experiment were performed for the plasmid construction, without any necessities for PCR, DNA purification, or gel electrophoresis. The construction could be completed within four hours with minimal lab work. The assembled plasmid from these 4 parts was illustrated in Fig. 3c.

### The development of genomic DNA editing protocol with one plasmid construction and one transformation

To develop a mature protocol for fellow researchers to use without further try and error, we went through the experiment process several times and optimized parameters to ensure successful genome editing. The protocol was divided in 4 steps. The first and second steps were to construct two modular parts, N20 part and donor DNA part. The third step was to assemble two ready-made modular parts with N20 and donor DNA parts with Golden Gate method in one reaction. The last step included one transformation and one culturing practice to obtain edited strains. The detailed protocol is presented in Additional file 1.

## Discussion

Compared to other CRISPR-based genome modification system, our one-plasmid system has the advantage of fast and easy operation with high efficiency. A simple comparison is summarized in Table 4 to illustrate difference of this technique compared with several recently developed methods. Information in this table is by no means accurate and subjective, and some description is by estimation. Due to the simplicity of one plasmid without any other supplemental DNA material, the experiment process is simple and less time consuming than other methods, only takes 3 days to complete. Also, labor intensity is relatively low, since major work only includes one plasmid construction and one transformation plus colony screening for editing plasmid and edited strains. We have experimented thoroughly to have selected optimized plasmid assembly strategy with ready-made parts (part1 and part2) and fixed linkers for editing plasmid construction. Because of this modularized design of plasmid construction, plasmid construction becomes very easy and economical. The construction process only takes 4 h after receiving synthesized DNA oligos, and minimal sets of oligos ensure low cost. The preferred editing protocol employed short homologous arms to achieve easiest plasmid construction but sacrificed some editing efficiency. In this comparison category, our method might have no advantage comparing to others (Table 4). However, at least one successfully edited strain could be obtained from seven to eight colonies. Furthermore, colony PCR screening is not laborious and is to be performed even with high editing efficiency experiments.

**Table 4:** Comparison of different methods using CRISPR/Cas9 system

	System composition	Transformation times	Cost	Editing efficiency	Time cost	Plasmid curing time	Labor intensity
pRed_Cas9_recA system this study	One plasmid	1	Low	13.8 ± 7.9%(41 bp homologous arms) 100%(more than300 bp homologous arms)	3 days (including plasmid construction, genome editing and plasmid curing)	1 day	Low
System established by Li [12]	Two plasmid and linear donr DNA	2	Medium	Around 100%	ND (estimated 4 days)	ND	Medium
System established by Jiang [11]	Two plasmid and/or linear donr DNA	2	Medium	Around 100%	4 days(without plasmid construction)	ND	Medium
System established by Jiang [10]	Two plasmid and/or linear donr DNA	2	Medium	65 ± 14%	ND (estimated more than 4 days without plasmid construction)	ND	Medium

*ND* not determine

The method also has an advantage for editing of multiple loci. The system requires only one temperature sensitive plasmid, which makes plasmid curing process easy and fast. In continuous process with our technique, X loci could be edited within 3× days. This rapid and simple method for *E. coli* genome editing has already been used in our research group, for example editing promoters of *crt*, *idi* and *dxs* in QL002 strain [23]. And it has also already been adopted by other labs in our institute.

High throughput and automatic genomic editing is one highly desired technique by molecular biologists [24, 25]. Due to its modular design, this method could be applied to develop automatic genome editing techniques. In plasmid construction process, DNA annealing and Golden Gate assembly process could be easily transformed to computer controlled practice with a liquid handler platform [26, 27]. Plasmid transformation and induction process could be automated as the MAGE system [24, 28]. Colonies PCR screening process can also be performed by automatic system [26, 27]. We expect a combined automatic system to be able to perform high-throughput, rationally designed genome editing based on our methods.

Colony PCR was used to identify successfully edited clones based on the size of PCR products in this work. However, as shown in Fig. 2c, some colony PCRs turned out to have neither edited band nor original band, just empty lanes. These colony PCRs were repeated with more sets of primers and were confirmed not to be able to obtain amplification products. A newly published article suggested that *E. coli* strains might possess a distinct end-joining activity that repairs double strand breaks (DSBs) and generate genome rearrangements. This mechanism, named alternative end-joining (A-EJ), is characterized by extensive DNA end restriction and large chromosomal loss [29]. We hypothesized that the colonies without PCR products might be *E. coli* cells that survived CRISPR mediated DSBs by A-EJ and underwent large DNA deletion. This hypothesis was partially proved by editing of *lacZ* locus. As illustrated in Table 3, 89% edited strains were white which apparently had *lacZ* knocked out. However, only 19% was confirmed to have shortened *lacZ* gel bands by colony PCR, which was the indication of precise editing as designed. Others showed no band or smear on gel, indicating changed chromosomal status around the *lacZ* locus. To analyze a larger area, primers targeting a region up to 7.5 kb around the Cas9 cleavage locus were designed and applied. However, no target PCR products were obtained with these primers either. Due to cost reason, we had not sequence these strains yet to find out their genomic changes. According to a newly published article, our result suggest CRISPR mediated DSBs may induce A-EJ repair in *E. coli*, and cause very large genomic deletion or rearrangement [30].

The method established in this paper has several advantages compared to others, however, its editing efficiency for short homologous arms did not reach 100%. There are some strategies we would exploit to improve in the near future. For example, using new CRISPR proteins, such as Cpf1 might help to improve recombination frequency by its different cleavage pattern [31]. Moreover, we might further improve homologous recombination efficiency by experimenting with more recombinases from various sources [32, 33].

## Conclusion

In this study, we achieved the goal of development of a very fast and easy genome editing technique with high efficiency based on CRISPR/Cas9 system that only required the work of one plasmid construction and one transformation, which allowed modification of a chromosome locus within 3 days and could be performed continuously for multiple loci.

## Additional files

**Additional file 1.** Standard genome editing protocol developed in this research.

**Additional file 2.** Plasmids profiles and sequences.

## References

[1] Yu D, Ellis HM, Lee E-C, Jenkins NA, Copeland NG (2000). An efficient recombination system for chromosome engineering in *Escherichia coli*. Proc Natl Acad Sci USA.

[2] Jantama K, Haupt M, Svoronos SA, Zhang X, Moore J, Shanmugam K, Ingram L (2008). Combining metabolic engineering and metabolic evolution to develop nonrecombinant strains of *Escherichia coli* C that produce succinate and malate. Biotechnol Bioeng.

[3] Datsenko KA, Wanner BL (2000). One-step inactivation of chromosomal genes in *Escherichia coli* K-12 using PCR products. Proc Natl Acad Sci USA.

[4] Bibikova M, Golic M, Golic KG, Carroll D (2002). Targeted chromosomal cleavage and mutagenesis in Drosophila using zinc-finger nucleases. Genetics.

[5] Miller JC, Tan S, Qiao G, Barlow KA, Wang J, Xia DF, Meng X, Paschon DE, Leung E, Hinkley SJ (2011). A TALE nuclease architecture for efficient genome editing. Nat Biotechnol.

[6] Mahfouz MM, Li L, Shamimuzzaman M, Wibowo A, Fang X, Zhu J-K (2011). De novo-engineered transcription activator-like effector (TALE) hybrid nuclease with novel DNA binding specificity creates double-strand breaks. Proc Natl Acad Sci USA.

[7] Horvath P, Barrangou R (2010). CRISPR/Cas, the immune system of bacteria and archaea. Science.

[8] Jakočiūnas T, Bonde I, Herrgård M, Harrison SJ, Kristensen M, Pedersen LE, Jensen MK, Keasling JD (2015). Multiplex metabolic pathway engineering using CRISPR/Cas9 in *Saccharomyces cerevisiae*. Metab Eng.

[9] Jinek M, Chylinski K, Fonfara I, Hauer M, Doudna JA, Charpentier E (2012). A programmable dual-RNA–guided DNA endonuclease in adaptive bacterial immunity. Science.

[10] Jiang W, Bikard D, Cox D, Zhang F, Marraffini LA (2013). RNA-guided editing of bacterial genomes using CRISPR-Cas systems. Nat Biotechnol.

[11] Jiang Y, Chen B, Duan C, Sun B, Yang J, Yang S (2015). Multigene editing in the *Escherichia coli* genome via the CRISPR-Cas9 system. Appl Environ Microbiol.

[12] Li Y, Lin Z, Huang C, Zhang Y, Wang Z, Tang YJ, Chen T, Zhao X (2015). Metabolic engineering of *Escherichia coli* using CRISPR–Cas9 meditated genome editing. Metab Eng.

[13] Shalem O, Sanjana NE, Hartenian E, Shi X, Scott DA, Mikkelsen TS, Heckl D, Ebert BL, Root DE, Doench JG (2014). Genome-scale CRISPR-Cas9 knockout screening in human cells. Science.

[14] Fujii W, Kawasaki K, Sugiura K, Naito K (2013). Efficient generation of large-scale genome-modified mice using gRNA and CAS9 endonuclease. Nucleic Acids Res.

[15] Chang N, Sun C, Gao L, Zhu D, Xu X, Zhu X, Xiong J-W, Xi JJ (2013). Genome editing with RNA-guided Cas9 nuclease in zebrafish embryos. Cell Res.

[16] Pyne ME, Moo-Young M, Chung DA, Chou CP (2015). Coupling the CRISPR/Cas9 system to lambda Red recombineering enables simplified chromosomal gene replacement in *Escherichia coli*. Appl Environ Microbiol.

[17] Benzinger R, Enquist LW, Skalka A (1975). Transfection of *Escherichia coli* spheroplasts. V. Activity of recBC nuclease in rec+ and rec minus spheroplasts measured with different forms of bacteriophage DNA. J Virol.

[18] Hillson NJ, Rosengarten RD, Keasling JD (2011). j5 DNA assembly design automation software. ACS Synth Biol.

[19] Ronda C, Pedersen LE, Sommer MO, Nielsen AT (2016). CRMAGE: CRISPR optimized MAGE recombineering. Sci Rep.

[20] De Mey M, De Maeseneire S, Soetaert W, Vandamme E (2007). Minimizing acetate formation in *E. coli* fermentations. J Ind Microbiol Biotechnol.

[21] Wang J, Sarov M, Rientjes J, Hu J, Hollak H, Kranz H, Xie Y, Stewart AF, Zhang Y (2006). An improved recombineering approach by adding RecA to λ red recombination. Mol Biotechnol.

[22] Engler C, Kandzia R, Marillonnet S (2008). A one pot, one step, precision cloning method with high throughput capability. PLoS ONE.

[23] Zhao J, Li Q, Sun T, Zhu X, Xu H, Tang J, Zhang X, Ma Y (2013). Engineering central metabolic modules of *Escherichia coli* for improving β-carotene production. Metab Eng.

[24] Wang HH, Isaacs FJ, Carr PA, Sun ZZ, Xu G, Forest CR, Church GM (2009). Programming cells by multiplex genome engineering and accelerated evolution. Nature.

[25] Jakočiūnas T, Jensen MK, Keasling JD (2016). CRISPR/Cas9 advances engineering of microbial cell factories. Metab Eng.

[26] Linshiz G, Stawski N, Poust S, Bi C, Keasling JD, Hillson NJ (2012). PaR-PaR laboratory automation platform. ACS Synth Biol.

[27] Linshiz G, Stawski N, Goyal G, Bi C, Poust S, Sharma M, Mutalik V, Keasling JD, Hillson NJ (2014). PR-PR: cross-platform laboratory automation system. ACS Synth Biol.

[28] Wang HH, Kim H, Cong L, Jeong J, Bang D, Church GM (2012). Genome-scale promoter engineering by coselection MAGE. Nat Methods.

[29] Chayot R, Montagne B, Mazel D, Ricchetti M (2010). An end-joining repair mechanism in *Escherichia coli*. Proc Natl Acad Sci USA.

[30] Cui L, David B (2016). Consequences of Cas9 cleavage in the chromosome of *Escherichia coli*. Nucleic Acids Res.

[31] Zetsche B, Gootenberg JS, Abudayyeh OO, Slaymaker IM, Makarova KS, Essletzbichler P, Volz SE, Joung J, van der Oost J, Regev A (2015). Cpf1 is a single RNA-guided endonuclease of a class 2 CRISPR-Cas system. Cell.

[32] Dudáš A, Chovanec M (2004). DNA double-strand break repair by homologous recombination. Mutat Res Rev Mut Res.

[33] Court DL, Sawitzke JA, Thomason LC (2002). Genetic Engineering Using Homologous Recombination 1. Annu Rev Genet.

