# Supplementary material for: Development of a fast and easy method for Escherichia coli genome editing with CRISPR/Cas9
Source: Microb Cell Fact. 2016 Dec 1;15:205. doi: 10.1186/s12934-016-0605-5 (PMC5134288; doi:10.1186/s12934-016-0605-5)
Supplement: Supplementary file 1 — Additional file 1. Standard genome editing protocol developed in this research. [file 12934_2016_605_MOESM1_ESM.docx]

**Supplementary Information**

**Development of a rapid and simple method for** ***Escherichia coli* genome edition** **with CRISPR/Cas9**

Dongdong Zhao, Shenli Yuan, Bin Xiong, Hongnian Sun, Lijun Ye, Jing Li, Xueli Zhang and Changhao Bi

**Standard genome editing protocol developed in this research**

**Step1: Selection of a target sequence**

The genomic target sequence should fulfill the following requirements:

20-nt long, and followed by a 3-nt PAM sequence: NGG, like 5'.. NNNNNNNNNNNNNNNNNNNN**NGG**..3'. Due to small genome size of *E. coli*, normally there is not necessary to considering off-target possibility.

**Step2: construction of N20 and donor DNA parts for plasmid construction**

(1) N20 part

The N20 could be easily constructed by annealing a pair of 24 nt oligos.

First, design oligos based on the following template:

sense oligo: 5'..AGCGNNNNNNNNNNNNNNNNNNNN..3'

antisense oligo: 3'.. NNNNNNNNNNNNNNNNNNNNCAAA..5'

The sense 20 nt oligo sequence is the target genomic sequence selected in step 1.

Then, phosphorylate oligos by the following experiment:

1 ul oligo I (100 uM)

1 ul oligo II (100 uM)

1ul 10X T4 Ligase buffer (NEB)

1 ul T4 PNK (NEB)

10 ul total volume

Finally, anneal oligos:

Place the mixture in a thermocycler using the follow in parameters to phosphorylate and anneal the oligos.

37℃ 30 min

95℃ 5 min and then ramp down to 25℃ at 5℃/min

4℃ hold until ready to proceed

Add 2μL of annealed oligos into 398 μl of water to dilute the products 200-fold.

(2) Construction of donor DNA part

A pair of 86nt oligos consisted of forward and reverse homologous arms and 4 nt overhangs are synthesized as the follow instruction. Then the two oligos are annealed to form donor DNA parts with the same protocol as annealing of the N20 parts above.

sense oligo: 5'.. GTGCN_1_ N_2_……N_82_..3' and

antisense oligo: 3'.. N_82_’N_81_’_…..._N1’CGAG.. 5', N1 to N41 is front homologous arm, and N42 to N82 is back homologous arm.

For genome insertion or replacement, two primers could be designed to insert amplification: 5'..CCAGGTCTCAGTGC - 41nt front homologous arm - 18~22nt forward primer of insertion part..3' and 5'..CCAGGTCTCAGAGC - 41nt back homologous arm - 18~22nt reverse primer of insertion part ..3'could be used for donor DNA PCR. The PCR product of this pair of primers are used for donor DNA part.

(3) The cloning of backbone modular part

Part1 and part2 are fixed parts, are directly amplified from pRed_Cas9_recA_△*poxb*300 and stored to construct plasmids targeting various loci.

**Step3: Plasmid assembly**

Add around 100 ng of part1 and equal molar amounts of the other assembly pieces to a 15 µl total volume assembly reaction mixture as follows:

Part1 and part2 (100 ng)

+ each additional assembly piece (to equal molar with backbone)

+ 1.5 µl 10X NEB T4 Buffer

+ 0.15 µl 100X BSA

+ 1 µl BsaI

+ 1 µl NEB T4 Ligase, 2 million cohesive end units / mL

+ dH20 to 15 µl

Perform the assembly reaction in a thermocycler as follows:

3 min @ 37 C }

4 min @ 16 C } 25 cycles

5 min @ 50 C }

5 min @ 80 C } 1 cycle

Specially designed fixed primer pairs are used for colony PCR to identify successfully assembled plasmids. Primers pkd_Cas9_0033F and pkd_Cas9_0033R can be used for the identification of donor DNA ligation, primers 0033_N20_F and 0033_N20_R can be used for the identification of N20 ligation.

**Step4: Procedure for genome editing**

Assembled plasmids are transformed into host by electroporation. The transformants carrying the editing plasmid are grown in 5 mL LB cultures with 50mg/L kanamycin at 30°C for 2 hours, 2g/L arabinose is added to the culture. Then culture is incubated more than 6 hours at 30°C. At last cells are diluted 100 fold and 20μL were plated on LB dishes containing 50mg/L kanamycin and 2g/L arabinose. Obtained colonies are identified by colony PCR with or without DNA sequencing.

**Step5: Plasmid curing and successive rounds of genome editing**

For the transformants with the expected genotype, editing plasmid could be cured by growing the colonies overnight at 37°C. The editing plasmid was found to be easy to be eliminated. The whole genome editing process including transformation, replacement and plasmid elimination normally took only 3 days, which is the fastest method for *E. coli* genome editing reported.

TableS1 The main primers used for this work

| Primers | Sequence | Characteristics |
| --- | --- | --- |
| poxb_genome _F | CGCCTTATGCCCGATGATATTC | colony PCR identification of *poxb* editing |
| poxb_genome _R | CCAGCACGCTGTTGTTAAAGAC |  |
| poxb_genome_long _F | AACTGTGCGGCGTAGTAG | colony PCR identification of *poxb* editing for 7.5kb area |
| poxb_genome_long _R | AAGCGCGGACATCGATAG |  |
| lacZ_genome_F | AAAACCCTGGCGTTACCCA | colony PCR identification of *lacz* editing |
| lacZ_genome_R | CAGGCAGTTCAATCAACTGTTTACC |  |
| part1_F | CCAGGTCTCAGCTCTGCTGAATGGAAGCTTGGATTCTCACC | Part1 amplication |
| part1_R | CCAGGTCTCACGCTTAAGATCTGACTCCATAACAGAGTACTCGCC |  |
| part2_F | CCAGGTCTCAGTTTTAGAGCTAGAAATAGCAAGTTAAAATAAGGC | part2 amplication |
| part2_R | CCAGGTCTCAGCACCACAGGCCCATGGATTCTTCG |  |
| pkd_cas9_0033F | GTGAAGGCCTGCATTATGTC | The identification of donor DNA ligation |
| pkd_cas9_0033R | CTCCACTTGCGTTAATAGGG |  |
| 0033_N20_F | AAGTACAGACAGGCGGATTC | The identification of donor N20 ligation |
| 0033_N20_R | CCTACCTACGTAACGGACTAAG |  |

TableS2 Oligonucleotides used for modular construction of pRed_cas9_recA_△*poxb*41, pRed_cas9_recA_△*poxb*::*rfp*41and pRed_cas9_recA_△*lacz*41

| Name | Oligonucleotide Sequence |
| --- | --- |
| N20_poxb_F | AGCGGCCGACACGT TAGTGCTACT |
| N20_poxb_R | AAACAGTAGCACTAACGTGTCGGC |
| N20_lacz_F | AGCGCTGGGGAATGAATCAGGCCA |
| N20_lacz_R | AAACTGGCCTGATTCATTCCCCAG |
| Donor DNA_poxb_F | GTGCGCTGGTTTCCAGCCCGGAGCAGATCCCACAAGTACTGGCGATCGACTCTGCGTGCATTGCTTCCATTGGTGGAAGAAAAAGC |
| Donor DNA_poxb_R | GAGCGCTTTTTCTTCCACCAATGGAAGCAATGCACGCAGAGTCGATCGCCAGTACTTGTGGGATCTGCTCCGGGCTGGAAACCAGC |
| Donor DNA_poxb::rfp_F | CCAGGTCTCAGTGC GCTGGTTTCCAGCCCGGAGCAGATCCCACAAGTACTGGCGA TGACGGCTAGCTCAGTCC |
| Donor DNA_poxb::rfp_R | CCAGGTCTCAGAGC GCTTTTTCTTCCACCAATGGAAGCAATGCACGCAGAGTCGA ACCCGAAGGTGAGCCAGTGTG |
| Donor DNA_lacz_F | GTGCTGAATTTGACCTGAGCGCATTTTTACGCGCCGGAGAAAACCGAAATGGTCCATCAAAAAATGGCTTTCGCTACCTGGAGAGA |
| Donor DNA_lacz_R | GTGCTGAATTTGACCTGAGCGCATTTTTACGCGCCGGAGAAAACCGAAATGGTCCATCAAAAAATGGCTTTCGCTACCTGGAGAGA |

**
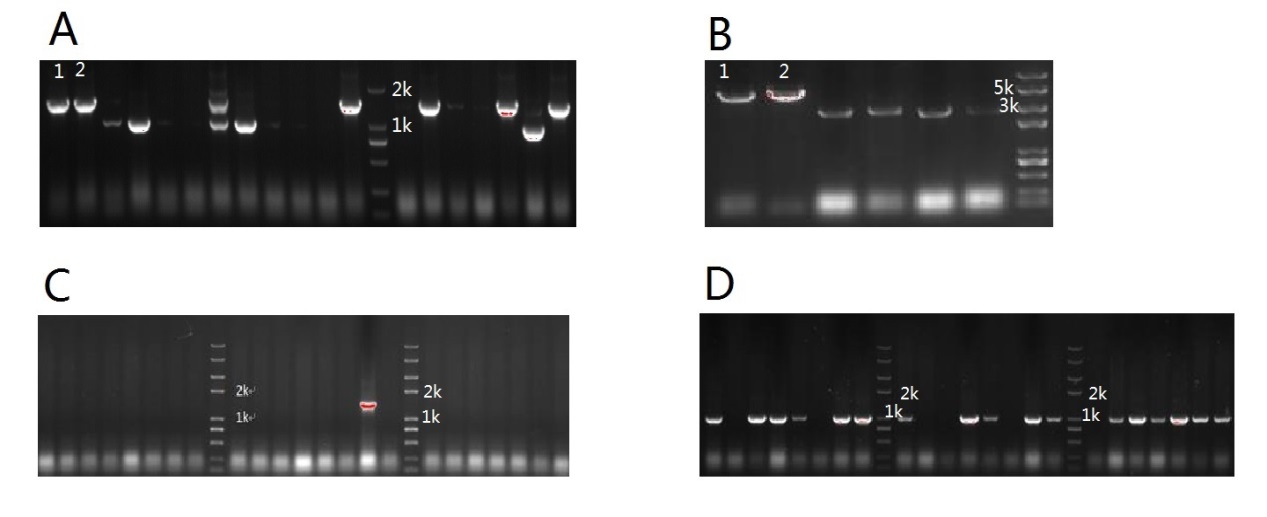
**

FigS1 Agarose gel electrophoresis of colony PCR for *poxb* edition using different plasmid

(A)*poxb* gene knock out with 513bp deletion using pAra_cas9_△*poxb*300: lane 1 and 2 are control group samples, others were experimental group samples. Colony PCR products of edited *poxb* gene were 1008p, and original were 1521bp. (B)*poxb* gene knock out with 1400bp deletion using pAra_cas9 series: lane 1 and 2 are control group samples, others were experimental group samples. Colony PCR products of edited *poxb* gene were 4281p, and original were 2880bp. (C) *poxb* gene knock out with 513bp deletion homologous arms of 51bp : all the bands were experimental group samples. Colony PCR products of edited *poxb* gene were 1008p. (D) and (C) *poxb* gene knock out with 513bp deletion homologous arms of 101bp : all the bands were experimental group samples. Colony PCR products of edited *poxb* gene were 1008p.
